# World Asthma Day 2026: asthma burden for patients and families will not be alleviated by science alone

**DOI:** 10.36416/1806-3756/e20260303

**Published:** 2026-08-13

**Authors:** Ricardo G Figueiredo, Regina Maria de Carvalho-Pinto

**Affiliations:** 1. Divisão de Pneumologia, Departamento de Saúde, Universidade Estadual de Feira de Santana - UEFS - Feira de Santana (BA) Brasil.; 2. Fundação ProAr, Salvador (BA) Brasil.; 3. Unidade de Doencas de Vias Aereas, Divisao de Pneumologia, Instituto do Coracao (InCor), Hospital das Clinicas HCFMUSP, Faculdade de Medicina, Universidade de Sao Paulo, Sao Paulo, SP, BR.

Asthma is one of the most common diseases in respiratory medicine and, surprisingly, one of the easiest to overlook. It affects children, adolescents, adults, and older people; it is seen in schools, emergency rooms, primary care clinics, and underserved rural areas. Recent global estimates suggest that about 260-300 million people live with asthma, and this number is likely to remain high as the population grows, ages, and moves into urban areas.^1^ ^,^ ^2^ Asthma is the most common chronic disease among children worldwide.^1^ In Brazil, the picture is equally concerning-according to available data, asthma prevalence is around 4.6% among adults and 12.1% among children, with asthma mortality of 1.32 deaths per 100,000 population.^3^ Population studies also show a high prevalence of asthma symptoms among adolescents, often around 19-23%, with significant regional heterogeneity and persistent gaps between symptoms, diagnosis, and treatment.^4^ ^,^ ^5^ Therefore, asthma should not be considered as a niche disease confined to specialists; it is a public health concern that impacts families, schools, workplaces, and healthcare systems every day.

Yet asthma remains neglected-not because it is rare, mysterious, or untreatable, but because its suffering is too often normalized. Wheezing becomes “bronchitis,” routinely managed in emergency settings with short-term systemic corticosteroids and short-acting bronchodilators, without further investigation or maintenance inhaled therapy. Nighttime symptoms are dismissed as “allergy.” Repeated emergency visits have become a normal part of families’ lives, impacting household income and productivity, especially among low-income families living with severe asthma.^6^ In global burden studies, chronic respiratory diseases continue to cause significant morbidity, mortality, and economic costs, with asthma remaining one of their leading contributors.^7^ In Brazil, national database analyses have documented a high number of asthma-related hospitalizations and deaths over recent decades, despite the availability of effective treatment and a universal public health system.^8^ ^-^ ^10^ This is the central scandal of asthma care: many severe attacks, hospitalizations, and deaths are potentially preventable. A fatal asthma attack is not always preceded by years of severe disease; sometimes it follows fragmented care, overreliance on bronchodilators, and the dangerous misconception that mild symptoms pose no risk.

For this reason, the GINA theme for World Asthma Day 2026-celebrated on May 5, the first Tuesday in May-is particularly apt: “Access to anti-inflammatory inhalers for everyone with asthma-still an urgent need.”^11^ The word “still” matters. It is a small word, but it carries decades of failure. We have known for many years that asthma is an inflammatory disease, not simply a problem of episodic bronchospasm. We also know that short-acting beta-agonists, while rapidly relieving symptoms, do not treat the underlying airway inflammation that drives exacerbation risk. The SABINA study raised concerns about short-acting β_2_ agonist (SABA) overprescription and over-the-counter availability, a pattern that may lead to poor control, recurrent need for oral corticosteroids, and increased risk of exacerbations and mortality.^12^ ^,^ ^13^ GINA’s message is not just about medication; it is practical and ethical. A health system that provides quick relief but not anti-inflammatory protection is offering incomplete and sometimes unsafe asthma care.

The modern asthma treatment paradigm has evolved significantly. GINA now emphasizes that everyone with asthma should receive ICS-containing therapy, either as regular preventive treatment or as an anti-inflammatory reliever strategy, depending on age, severity, availability, and clinical circumstances.^14^ In adults and adolescents, the preferred “Track 1” approach-using low-dose inhaled corticosteroid + formoterol as the reliever across treatment steps, and as maintenance-and-reliever therapy when needed-has shifted asthma management away from SABA-only approaches.^15^ This is not a cosmetic change in guidelines-it is a fundamental shift in treatment strategy: every symptom relief opportunity should also be an opportunity to treat inflammation. Evidence from studies (START, BATURA) and recent network meta-analyses consistently shows that anti-inflammatory reliever strategies reduce severe exacerbations compared with SABA alone.^16^ ^-^ ^18^ The evidence now clearly indicates that bronchodilator-only rescue is no longer an acceptable treatment for asthma management.

Inhaled corticosteroids are among the most important medications in respiratory medicine for the treatment of some airway diseases, especially asthma; however, they should not be romanticized. At low-to-moderate doses, their benefit-to-risk profile is favorable and far safer than repeated oral corticosteroid bursts. Still, dose as well as treatment duration matter for all patients, and children deserve vigilance. Endocrine effects of inhaled corticosteroids, including potential effects on growth and adrenal-axis function, have been well described, particularly at higher doses. Metabolomic data have also shown dose-related adrenal suppression signals in patients using inhaled corticosteroids, with measurable changes even at lower doses.^19^ The appropriate conclusion is not steroid fear-it is steroid stewardship: the appropriate dose, in the right device, for the right patient, with regular review and step-down when control is sustained.

The article by Albuquerque et al. in the current issue of the *Respiratory Research & Clinical Practice* adds another important layer to this discussion.^20^ In an exploratory study of 44 adolescents with asthma, the authors evaluated endogenous metabolites in exhaled breath condensate before and after eucapnic voluntary hyperventilation. Exercise-induced bronchoconstriction (EIB) was defined by a fall in FEV_1_ of at least 10% after challenge. The study found a clear physiological contrast: the mean decrease in FEV_1_ was much greater in adolescents with EIB than in those without. Notably, after ventilatory stress, orthogonal partial least squares discriminant analysis revealed a distinct separation of metabolic profiles among participants with EIB, a pattern that was not evident in those without EIB. Acetate, identified by a proton nuclear magnetic resonance signal at 1.76 ppm, emerged as a key differentiator.

This is not yet a practice-changing biomarker study, and the authors were appropriately cautious. The sample is small, the design is exploratory, and metabolomic signatures require external validation before they can shape routine care. But the study is valuable because it asks the right question. Exercise-induced bronchoconstriction is often treated as a simple clinical label: symptoms with exercise, confirmed or not by challenge testing, treated with reliever medication, and sometimes tolerated as a limitation. Adolescents, however, experience EIB in a deeply personal way. It may determine who joins a football game, who avoids physical education, who feels different, who becomes less active, and who gradually accepts breathlessness as part of their identity. A noninvasive metabolic window into EIB could help us understand why some young people with asthma respond to exertional stress with marked bronchoconstriction while others do not. That is precision medicine in its most useful form: not expensive technology for its own sake, but a better explanation of a real clinical problem.

The bridge between GINA’s World Asthma Day message and the Albuquerque et al. study might not be immediately obvious, but it is crucial. Access and innovation must not compete. In asthma care, we need both. It is hard to justify discussions about metabolomics, biologics, digital inhalers, artificial intelligence, or treatable traits while many patients still cannot access, afford, or properly use a corticosteroid-containing inhaler. At the same time, access to inhaled corticosteroids should be a cornerstone of every asthma policy. Public health must ensure the basics are available; research should aim to make care more precise, less burdensome, and more tailored to patients’ biology and lives. The future of asthma treatment should not be a choice between a basic inhaler for most and biologic treatments for the few. Notably, despite the increasing burden of respiratory diseases, public awareness remains limited, and researchers continue to face chronic underfunding and lack of institutional support compared to other highly prevalent conditions, such as cardiovascular and metabolic disorders and cancer.^21^

**Figure 1:**
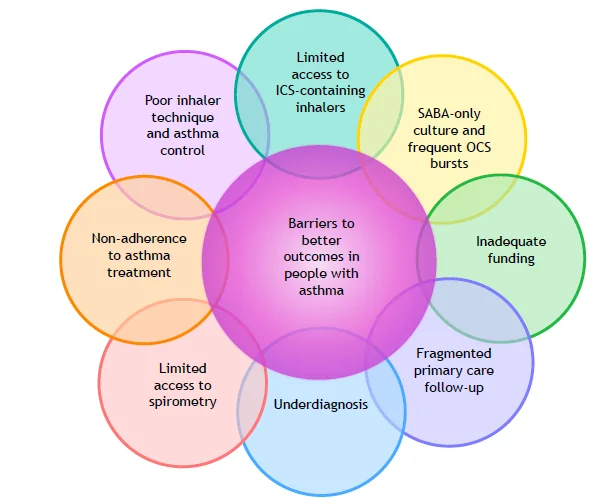
Asthma outcomes are shaped by access, implementation, education, and continuity of care. ICS: inhaled corticosteroid; SABA: short-acting β_2_ agonist; and OCS: oral corticosteroid.

Brazil is a particularly important setting for this conversation. The country has scientific capacity and a universal public health system, but it still faces persistent inequities in access, diagnosis, follow-up, and referral for severe asthma (Figure 1). Although beclomethasone metered-dose inhalers and salbutamol are available free of charge through the *Farmácia Popular* program, access to inhaled corticosteroid + formoterol and essential asthma care remains bureaucratic and uneven, particularly for socially vulnerable populations; meanwhile, oral corticosteroids are widely available without prescription, and the cumulative corticosteroid burden remains underestimated. A national asthma policy should be explicit, measurable, and integrated. It should start in primary care with diagnosis, access to spirometry, inhaler technique, and written action plans. It should include school-based strategies for children and adolescents, especially those whose symptoms limit exercise and social participation. It should establish clear referral pathways for difficult-to-treat and severe asthma, with access to specialist assessment, phenotyping, biologics when indicated, and vigilance regarding oral corticosteroid exposure. 

The Global Asthma Report has long emphasized that effective asthma care depends not only on medicines but also on surveillance, implementation, training, and access to quality-assured essential treatment.^22^ Brazilian experience also suggests that organized asthma control programs can reduce the human and economic burden of disease when access, education, and structured care are prioritized as policy rather than isolated clinical interventions.^23^ World Asthma Day 2026 should therefore be treated not as a commemorative date, but as a call for public health action on asthma. Asthma needs a national plan with a budget, governance, indicators, and accountability. Policymakers should strengthen primary care for diagnosis and longitudinal management, expand access to spirometry, and organize referral pathways from primary care to severe asthma reference centers. Payers and pharmaceutical manufacturers must also be part of the solution by keeping anti-inflammatory inhalers continuously available and financially sustainable. Research should continue to advance metabolomics, biomarkers, digital monitoring, severe asthma registries, and precision phenotyping, but always in the service of a broader public health agenda. Asthma has taught us enough pathophysiology to act. The challenge now is to turn that knowledge into policies that prevent attacks, hospitalizations, deaths, and the underestimated burden carried by patients and families.
